# A transcriptome profile in hepatocellular carcinomas based on integrated analysis of microarray studies

**DOI:** 10.1186/s13000-016-0596-x

**Published:** 2017-01-13

**Authors:** Feifei Wang, Ruliang Wang, Qiuwen Li, Xueling Qu, Yixin Hao, Jingwen Yang, Huixia Zhao, Qian Wang, Guanghui Li, Fengyun Zhang, He Zhang, Xuan Zhou, Xioumei Peng, Yang Bian, Wenhua Xiao

**Affiliations:** 1 Department of Oncology, The First Affiliated Hospital of PLA General Hospital, Fucheng Road 51, Beijing, 100048 China; 2 Department of Bioinformatics, Beijing Medintell Biomed Co., Ltd, Beijing, China

**Keywords:** Hepatocellular cancer, Differentially expressed gene, Integrated analysis, Expression profile, Real time polymerase chain reaction, TCGA validation

## Abstract

**Background:**

Despite new treatment options for hepatocellular carcinomas (HCC) recently, 5-year survival remains poor, ranging from 50 to 70%, which may attribute to the lack of early diagnostic biomarkers. Thus, developing new biomarkers for early diagnosis of HCC, is extremely urgent, aiming to decrease HCC-related deaths.

**Methods:**

In the study, we conducted a comprehensive characterization of gene expression data of HCC based on a bioinformatics method. The results were confirmed by real time polymerase chain reaction (RT-PCR) and TCGA database to prove the credibility of this integrated analysis.

**Results:**

After integrating analysis of seven HCC gene expression datasets, 1167 differential expressed genes (DEGs) were identified. These genes mainly participated in the process of cell cycle, oocyte meiosis, and oocyte maturation mediated by progesterone. The results of experiments and TCGA database validation in 10 genes was in full accordance with findings in integrated analysis, indicating the high credibility of our integrated analysis of different gene expression datasets. *ASPM*, *CCT3*, and *NEK2* was showed to be significantly associated with overall survival of HCC patients in TCGA database.

**Conclusion:**

This method of integrated analysis may be a useful tool to minish the heterogeneity of individual microarray, hopefully outputs more accurate HCC transcriptome profiles based on large sample size, and explores some potential biomarkers and therapy targets for HCC.

**Electronic supplementary material:**

The online version of this article (doi:10.1186/s13000-016-0596-x) contains supplementary material, which is available to authorized users.

## Background

Hepatocellular carcinoma (HCC) is one of the most frequently occurring malignant tumors worldwide [1]. Risk factors of HCC are well recognized including gender, infection by hepatitis B virus or hepatitis C virus, cirrhosis, metabolism diseases, toxins, excess alcohol consumption, and smoking. HCC varies with wide geography, and is more prevalent in Asia, Africa, and southern Europe. It has been well defined that experiencing surgery for early HCC patients could achieve a higher curative resection rate (80.5%) [2], and finally have a better survival rate. However, patients with early HCC frequently manifest non-typical symptoms, hence, most of patients are diagnosed with advanced HCC when seeing a doctor, resulting in a low 5-year survival rate, ranging from 50 and 70% [3]. Therefore, developing biomarkers for early diagnosis is being emphasized to prolong survival in patients with HCC.

Over the last decades, large efforts have been made to promote the early diagnosis of HCC. Alpha-fetoprotein (AFP) has been the most commonly used tumor biomarker in the liver, testicles, and ovaries [4]. Highly sensitive and specific biomarkers need to be developed in HCC diagnosis. Glypican-3 (GPC3), a membrane-associated heparan sulfate proteoglycan, is up-regulated in HCC. Additionally, GPC3 involved in hippo pathway to exert its function in HCC cell proliferation. GPC may be applied in clinical practice as a novel diagnostic biomarker [5].

Additionally, some researchers have attempted to employ prognostic markers for predicting HCC recurrence. Villa E et al. detected whole genome microarray expression profiling of 161 HCC samples, and revealed that five-gene signature (*ANGPT2*, *NETO2*, *NR4A1*, *DLL4*, *ESM1*) was able to predict fast growth and worst survival of HCC patients [6]. The exploration of prognostic markers may facilitate individualized therapies.

Recently, detection of genome-wide gene transcripts expressed in a given tissue type is becoming more and more feasible with advent of high-throughput technologies, such as microarray and RNA-seq. The application of microarray-based gene expression profiling has produced tremendous information, and provided mechanistic insights into the oncogenic process of HCC [7]. However, although many microarray studies of HCC have been performed [8–11], each of study holds a somewhat different view due to the heterogeneity caused by the variety in clinical samples, platform, analytical approach, etc. Toward this end, an integrated analysis of seven HCC gene expression datasets was conducted to identify differential expressed genes (DEGs) between tumor and normal tissues, revealing a common biological thread that linked the disparate microarray studies. Ten genes were selected for further real time polymerase chain reaction (RT-PCR) and TCGA database validation, to prove the credibility of this integrated analysis. We expected our study would be of some value for the future diagnosis and therapy of HCC in clinic.

## Methods

### Eligible HCC gene expression datasets

The raw gene expression datasets of HCC and control samples were selected and downloaded in the Gene Expression Omnibus (GEO) database. The datasets meeting the following criteria were included: i) the expression profile of whole genome; ii) data from the tumor and tumor-adjacent normal liver tissues from HCC patients in clinic; iii) raw data or standardized data. Cirrhotic liver tissue sets, non-human sets, and integrated analysis of gene expression profiles were excluded.

### Identification of HCC gene expression profile

We selected the Z-score transformation [12] method to normalize raw data from different platforms. The MATrixLABoratory (MATLAB) software was applied to calculate differently expressed probe sets between tumor and tumor-adjacent normal tissue, using gene specific *t*-test. The genes with FDR ≤ 0.05 were selected as the significantly differentially expressed genes (DEGs). Heat map analysis was conducted using the “heatmap.2” function of the R/Bioconductor package “gplots” [13].

### Gene ontology (GO) of differentially expressed genes

The GO and pathway enrichment was analyzed via the online software GENECODIS to facilitate the interpretation of biological roles of DEGs (http://genecodis.cnb.csic.es) [14]. The GO functions of the DEGs were determined according to different categories including biological process, molecular functions, and cellular components. In addition, pathway enrichment analysis was based on the Kyoto Encyclopedia of Genes and Genomes (KEGG) database.

### Protein-protein interaction (PPI) network construction

In order to find candidate genes involved in the oncogenesis and hepatic dysfunction of HCC, PPI networks of significantly DEGs were constructed according to the data from Biological General Repository for Interaction Datasets (BioGRID) (http://thebiogrid.org/). Among the candidate genes, the PPI networks of the top 20 most significantly dysregulated genes were visualized via Cytoscape [15].

### RNA Isolation and RT-PCR validation

Tumor and matched adjacent normal liver tissues which were obtained from five HCC patients in the current study, were frozen immediately after surgery, and were stored at −135 °C for RNA extraction. Frozen sections were made and evaluated independently by senior pathologists. The study was approved by the First Affiliated Hospital of PLA General Hospital ethnics committee. The ethics committee approved the relating screening, inspection, and data collection of the patients, and all subjects signed a written informed consent form. All works were undertaken following the provisions of the Declaration of Helsinki.

The whole RNA of liver tissue for each sample was extracted using RNAeasy Mini Kit (Qiagen, Valencia, CA) according to the manufacture’s protocol. Ten genes were randomly selected from the 20 most significantly DEGs. Primers for the ten genes were designed using PrimerPlex 2.61 (PREMIER Biosoft, Palo Alto, CA) (Additional file 1: Table S1). Expression levels of genes were screened by SYBR (Applied Biosystems/Life Technologies, Carlsbad, CA) in ABI 7500 Real Time PCR System (Applied Biosystems, Carlsbad CA). Relative gene expression was calculated with Data Assist Software version 3.0 (Applied Biosystems/Life Technologies) and human actin gene was used as a reference. The expression level of each gene was determined according to the method of 2^-△△ct^.

### TCGA database validation of selected genes in HCC patients

Through the online validation tools, the expression status of selected genes in HCC were determined in TCGA database (https://genome-cancer.ucsc.edu/), assessing their mRNA expression patterns in HCC patients (*N* = 423) [16]. The selected genes were also evaluated for the overall survival time of HCC patients in correlation with their expression pattern (http://cbioportal.org) in the TCGA database (*N* = 442) [17].

## Results

### Candidate genes involved in the occurrence of HCC

**Fig. 1:**
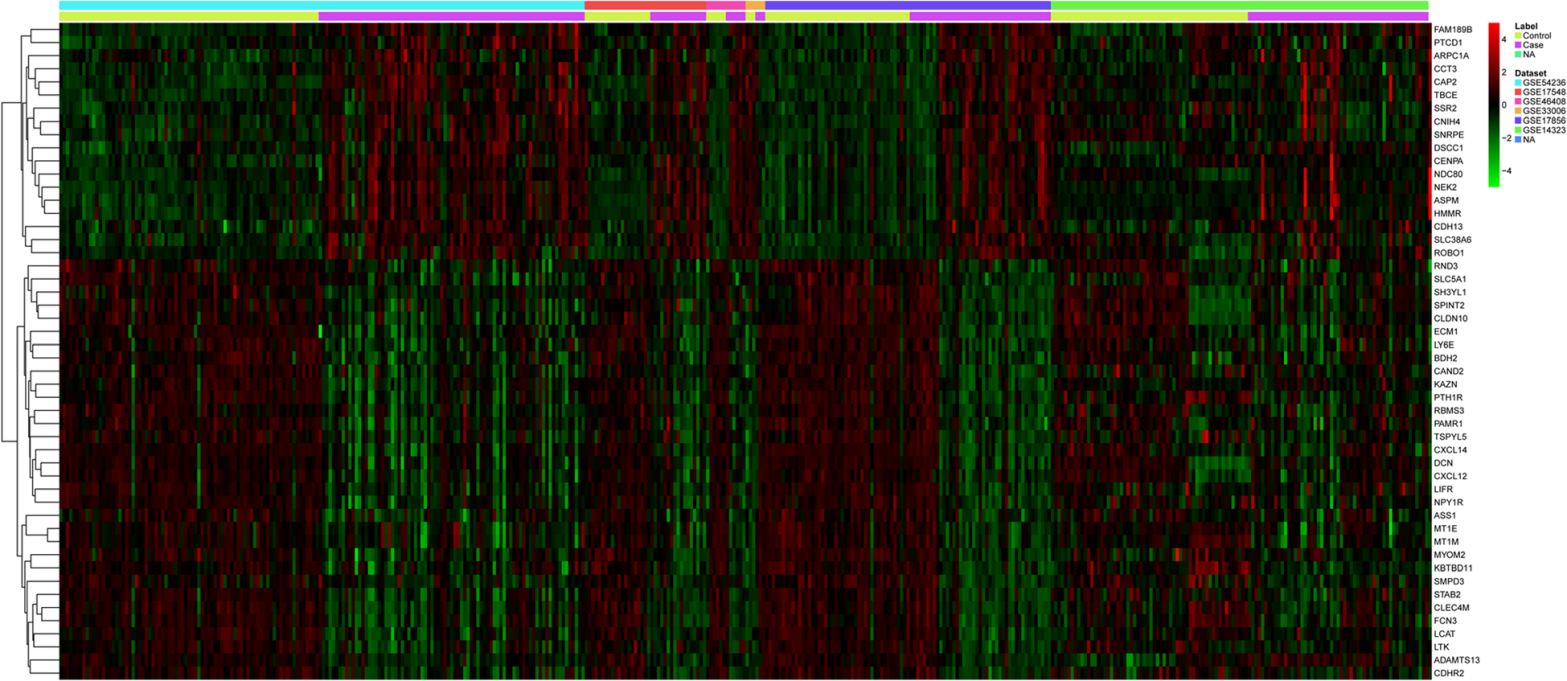
Heat-map image of the top 50 significantly up-regulated or down-regulated genes in HCC

Seven microarray datasets of HCC were identified according to the including criteria. Among of them, GSE17548, GSE33006, GSE17856, and GSE1481 didn’t contain the gene expression data of tumor-adjacent normal liver tissues. 267 HCC samples and 67 control samples were enrolled in the integrated analysis. The information of each microarray dataset was shown in Table 1. Based on microarray datasets available for integrated analysis, a total of 1167 DEGs were identified, among which, 628 genes were up-regulated and 539 genes were down-regulated. The detailed information of the 20 most significantly up-regulated or down-regulated genes were shown in Additional file 1: Table S2. The top 50 most significantly DEGs were displayed in a heat map across different HCC microarray datasets (Fig. 1).

**Table 1:** Information of the expression profiles

GEO ID	Platform	Samples (cancer:normal)	Sample source	Country	Time
GSE54236	GPL6480 Agilent-014850 Whole Human Genome Microarray 4x44K G4112F (Probe Name version)	64:19	In vivo	Italy	2014
GSE17548	GPL570 [HG-U133_Plus_2] Affymetrix Human Genome U133 Plus 2.0 Array	17:0	In vivo	Turkey	2013
GSE46408	GPL4133 Agilent-014850 Whole Human Genome Microarray 4x44K G4112F (Feature Number version)	6:6	In vivo	Taiwan	2013
GSE33006	GPL570 [HG-U133_Plus_2] Affymetrix Human Genome U133 Plus 2.0 Array	3:0	In vivo	Taiwan	2011
GSE17856	GPL6480 Agilent-014850 Whole Human Genome Microarray 4x44K G4112F (Probe Name version)	43:0	In vivo	USA	2010
GSE14811	GPL8177 KRIBB_Human_14K	56:0	In vivo	Korea	2009
GSE14323	GPL96 [HG-U133A] Affymetrix Human Genome U133A Array/GPL571 [HG-U133A_2] Affymetrix Human Genome U133A 2.0 Array	81:43	In vivo	USA	2009

### GO and KEGG analysis of the candidate genes

Mitotic cell cycle (GO: 0000278, 4.71E-36) and cell division (GO: 0051301, 7.83E-26) was significantly enriched upon the category of GO biological progress, and protein binding (GO: 0005515, 3.88E-85) and nucleotide binding (GO: 0000166, 2.71E-41) was significantly enriched upon the category of GO molecular function. While for the category of GO cellular component, cytoplasm (GO: 0005737, 1.77E-90) and nucleus (GO: 0005634, 5.20E-59) was significantly enriched (Table 2). Based on KEGG database, the 1167 DEGs were involved in 99 signal pathways, including cell cycle, oocyte meiosis, oocyte maturation mediated by progesterone, pathways in cancer, p53 signaling pathways, production of phagosome, metabolism of fatty acid, cytokines-cell factor receptor interactions, prion diseases, etc. (Table 3).

**Table 2:** Partial results of gene ontology (GO) analysis

GO ID	GO term	No.of genes	*F.D.R*
Biological process			
GO:0000278	mitotic cell cycle	71	4.71E-36
GO:0051301	cell division	58	7.83E-26
GO:0000087	M phase of mitotic cell cycle	33	4.04E-22
GO:0007049	cell cycle	65	1.37E-21
GO:0000236	mitotic prometaphase	30	2.02E-20
GO:0007067	mitosis	39	1.04E-17
GO:0007165	signal transduction	101	8.36E-16
GO:0000086	G2/M transition of mitotic cell cycle	25	1.82E-11
GO:0006260	DNA replication	27	2.81E-10
GO:0000075	cell cycle checkpoint	25	3.30E-10
GO:0007155	cell adhesion	54	5.13E-10
GO:0000082	G1/S transition of mitotic cell cycle	25	2.88E-09
GO:0006915	apoptotic process	54	5.52E-09
GO:0008285	negative regulation of cell proliferation	38	1.49E-08
GO:0007596	blood coagulation	44	5.51E-08
Molecular function			
GO:0005515	protein binding	405	3.88E-85
GO:0000166	nucleotide binding	203	2.71E-41
GO:0005524	ATP binding	147	1.90E-30
GO:0046872	metal ion binding	171	7.35E-12
GO:0016301	kinase activity	30	2.35E-08
GO:0003824	catalytic activity	38	1.41E-07
GO:0016787	hydrolase activity	69	3.22E-07
GO:0016491	oxidoreductase activity	41	3.62E-07
GO:0009055	electron carrier activity	24	3.88E-07
GO:0019901	protein kinase binding	28	6.24E-07
GO:0003677	DNA binding	103	2.55E-06
GO:0004672	protein kinase activity	29	4.58E-06
GO:0019899	enzyme binding	23	6.10E-06
GO:0004674	protein serine/threonine kinase activity	34	9.76E-06
GO:0008017	microtubule binding	14	1.00E-05
Cellular component			
GO:0005737	cytoplasm	455	1.77E-90
GO:0005634	nucleus	403	5.20E-59
GO:0005829	cytosol	214	3.68E-47
GO:0005654	nucleoplasm	99	4.43E-24
GO:0005730	nucleolus	129	2.06E-22
GO:0005694	chromosome	45	1.28E-17
GO:0005576	extracellular region	141	1.74E-17
GO:0005615	extracellular space	77	1.18E-15
GO:0005886	plasma membrane	206	4.70E-14
GO:0005856	cytoskeleton	77	6.31E-14
GO:0016020	membrane	221	1.36E-12
GO:0005819	spindle	25	1.40E-12
GO:0000777	condensed chromosome kinetochore	18	2.41E-11
GO:0005874	microtubule	34	4.10E-10
GO:0005622	intracellular	122	4.20E-10

**Table 3:** Partial results of Kyoto Encyclopedia of Genes and Genomes (KEGG) analysis

KEGG ID	KEGG term	No. of genes	FDR
hsa04110	Cell cycle	29	1.08E-14
hsa04114	Oocyte meiosis	20	5.61E-08
hsa04914	Progesterone-mediated oocyte maturation	17	1.93E-07
hsa05200	Pathways in cancer	33	6.75E-07
hsa04115	p53 signaling pathway	13	1.07E-05
hsa04145	Phagosome	18	1.75E-05
hsa00071	Fatty acid metabolism	10	1.98E-05
hsa04060	Cytokine-cytokine receptor interaction	26	2.02E-05
hsa05020	Prion diseases	9	2.65E-05
hsa00230	Purine metabolism	19	2.73E-05
hsa00830	Retinol metabolism	11	8.59E-05
hsa04360	Axon guidance	16	9.27E-05
hsa00590	Arachidonic acid metabolism	10	1.20E-04
hsa05110	Vibrio cholerae infection	10	1.20E-04
hsa00240	Pyrimidine metabolism	13	1.45E-04

### PPI Network Constructions

**Fig. 2:**
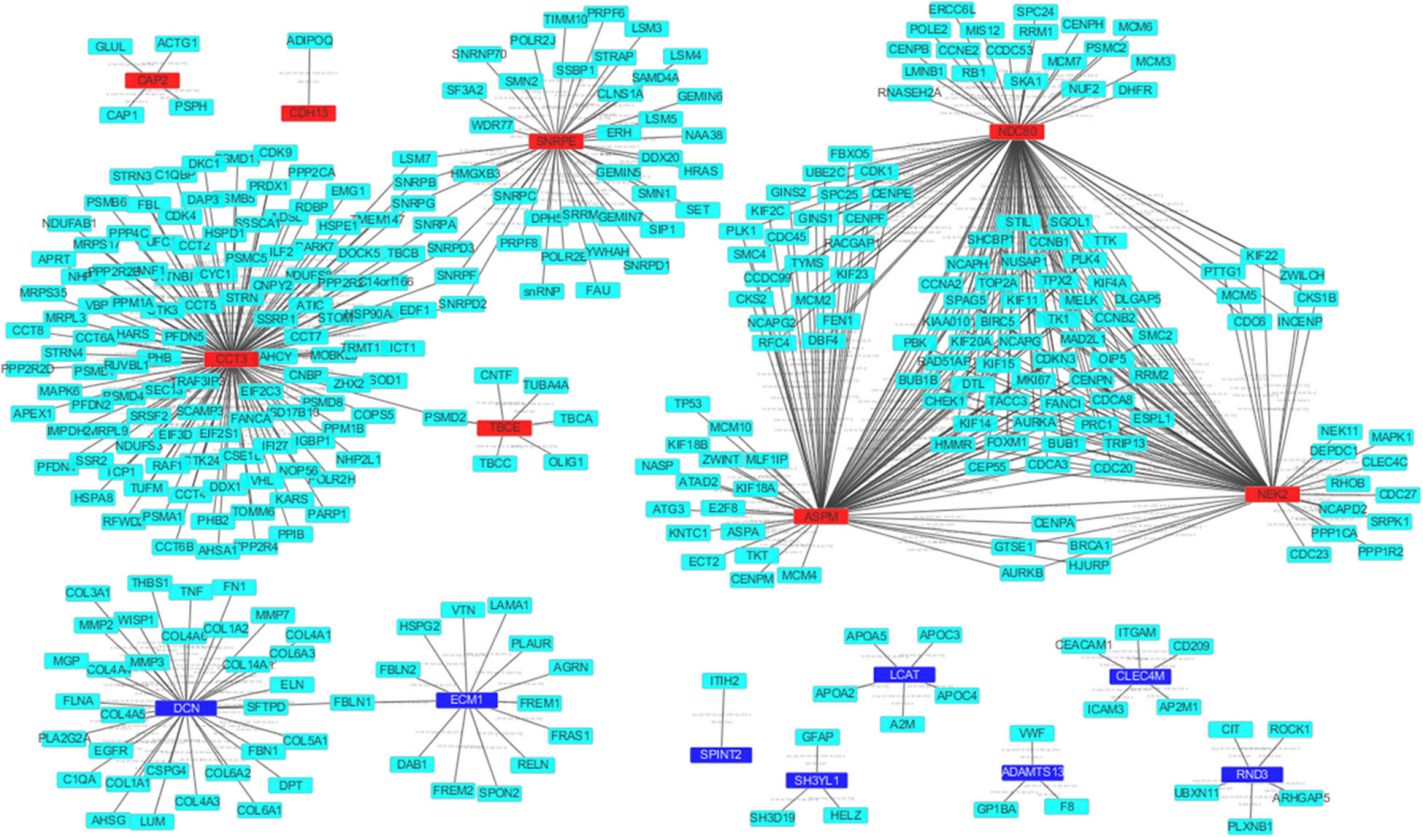
Protein-protein interaction analysis of the 20 most significantly DEGs: Red was up-regulated DEGs; Blue was down-regulated DEGs

For PPI networks of the 20 most significantly dyregulated genes, they consisted of 377 edges and 503 nodes. Three hub proteins were identified in this network, including *CCT3* (121°), *NDC80* (98°), and *ASPM* (93°) (Fig. 2).

### Experimental and TCGA database validation of selected genes in HCC patients

**Fig. 3:**
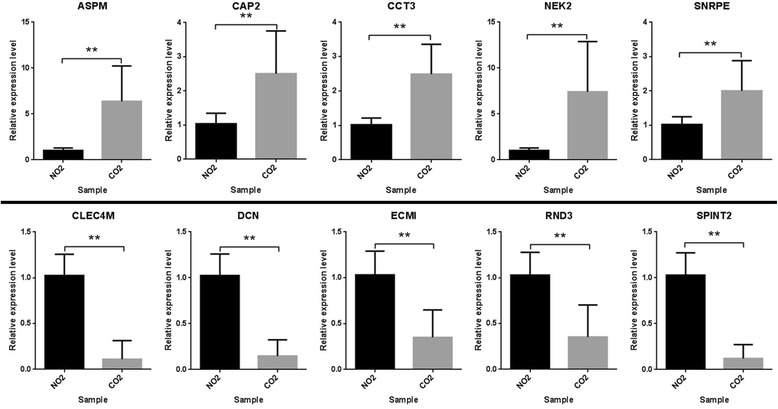
RT-PCR validation in HCC clinical samples for mRNA expression level of 10 most significantly dysregulated genes. NO2; control samples; CO2; HCC samples. **; significant difference with *P* < 0.01

**Fig. 4:**
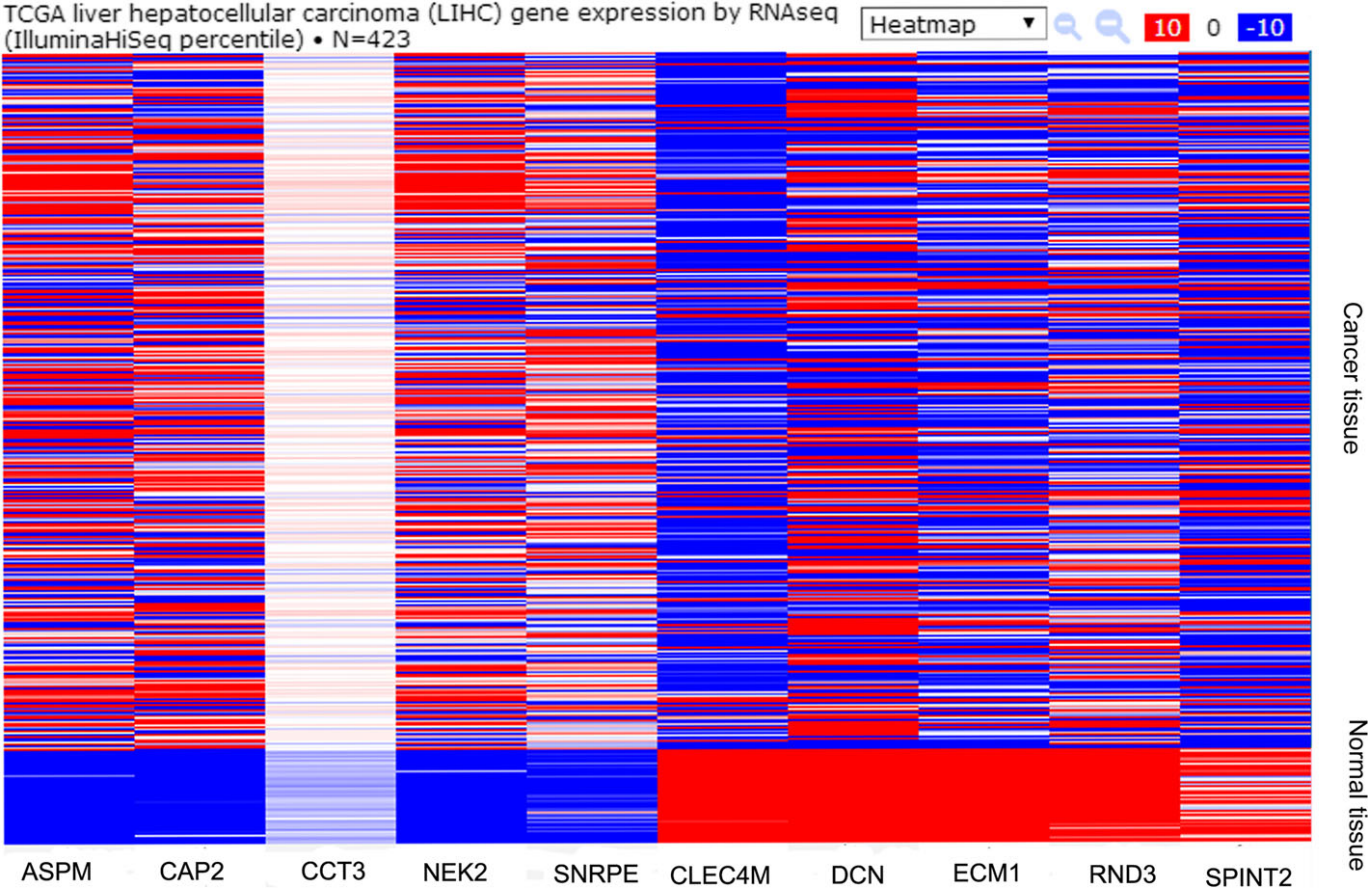
TCGA database validation for mRNA expression level of 10 most significantly dysregulated genes

**Fig. 5:**
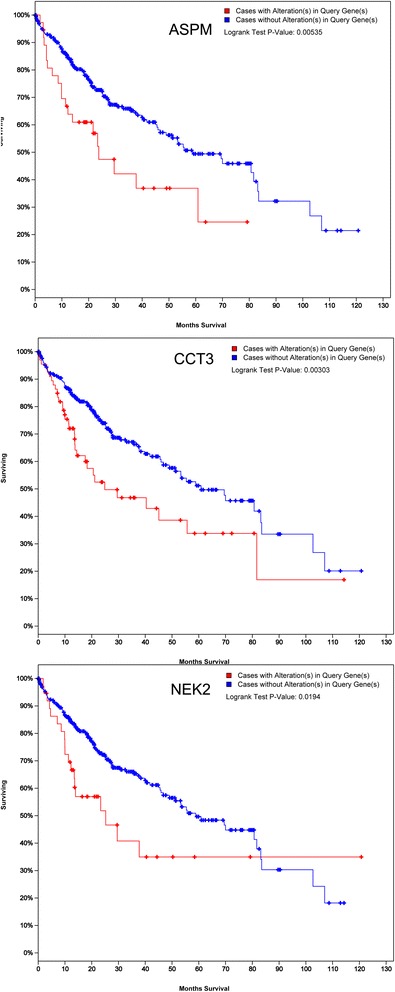
The association between gene expression level and HCC survival in TCGA database for 10 most significantly dysregulated genes

Ten genes (*ASPM, CAP2, CCT3, NEK2, SNRPE, CLEC4M, DCN, ECM1, RND3* and *SPINT2*) were randomly retrieved from the 20 most significantly up-regulated or down-regulated genes, respectively. After performing RT-PCR, the expression levels of selected 10 genes in clinical samples were identical with the results of the integrated analysis. For the ten genes, the mRNA expression was statistically different between tumor and matched adjacent normal liver tissues (Fig. 3; Additional file 1: Table S3) (*P* < 0.01). Furthermore, results of TCGA database validation indicated that these genes showed similar expression trends to those obtained from the integrated analysis (Fig. 4). Among the ten genes, only the ASPM, CCT3, and NEK2 showed significant association with overall survival time of HCC patients in TCGA database (*P* < 0.05) (Fig. 5).

## Discussion

It is generally accepted that the altered gene expression pattern of a cancer tissue should be associated with the initiation and maintenance of the malignant phenotype. Previous studies have identified several HCC gene expression profiles [18–21]. However, there wasn’t a common pattern among disparate studies for HCC. While in this study, we integrated different microarray studies to identify a precise gene expression profile for HCC with more statistical power supported by large sample size. In the current study, an integrated analysis of seven HCC microarray datasets was conducted, and showed that 1167 DEGs were identified, among which 628 genes were up-regulated and 539 genes were down-regulated. These genes mainly participated in the process of cell cycle, oocyte meiosis, and oocyte maturation mediated by progesterone.

In the current study, further annotation and PPI network analysis of the 20 most significant DEGs were conducted. Most of the 20 genes were involved in the pathways of cell cycle, cytokines-cell factor receptor interactions, and intracellular signaling cascades, and their involvements in HCC have also been reported [22–26]. The functions of the 20 genes were in accordance with the results of GO and KEGG analysis. Three genes, including *CCT3*, *NDC80*, and *ASPM* were proved to be highly connected in the PPI network. *CCT3,* a subunit of CCT cluster, plays a role in assisting the folding of proteins involved in important biological processes. *CCT3* was found to display a significantly different gene expression level in HCC compared to adjacent non-malignant liver tissues, arising from the occurrence of the amplicon 1q21-q22 [27], which is consistent with our result of RT-PCR validation. In addition, other genes’ expression status detected by RT-PCR was totally in accordance with the result of integrated analysis, suggesting that the bioinformatics method of integrated analysis was credible.

*ASPM* was highly expressed in fetal tissues but lowly in most adult tissues. Our result and previous evidences [23] found that *ASPM* and *NEK2* mRNA was over-expressed in HCC. Moreover, we found that *ASPM*, *NEK* and *CCT3* over-expression present significant association with overall survival of HCC patients based on TCGA validation, predicting enhanced invasive/metastatic potential of HCC and higher risk of early tumor recurrence. *ASPM*, *NEK* and *CCT3* may be applied as potential prognostic biomarkers for HCC. *CAP2* overexpression was also discovered in our study, and *CAP2* has been suggested as a candidate biomarker of HCC owing to elevated level in the serum of HCC patients [28].

Among the 10 most significantly down-regulated genes, *DCN*, an extracellular matrix proteoglycan, has important biological functions in growth, development and diseases. Loss of the decorin gene, which are known to interfere with cellular events of tumorigenesis mainly by blocking various receptor tyrosine kinases such as EGFR, Met, IGF-IR, PDGFR and VEGFR2, is permissive for tumorigenic growth of HCC with decreasing levels of the cyclin-dependent kinase inhibitor p21^*WAF1/CIP1*^, suggesting potential utilization of *DCN* as an antitumor agent in HCC [29]. *RND3* down-regulation in HCC patients has been reported by several studies [26, 30, 31], and may be a metastasis suppressor gene in HCC.

However, the expression patterns of four genes among the 20 most significant DEGs in the current study were inconsistent with or ignored in the previous studies, including *TBCE*, *SPINT2*, *ECM1*, and *KZAN*. The function of *KZAN* was not identified, whereas the other three genes were all comprehensively studied. In the current study, the inconsistent results might inspire their roles in the oncogenesis and development of HCC with some novel views.

*SPINT2* encodes a transmembrane protein with two extracellular Kunitz domains that inhibits a variety of serine proteases. The protein product of *SPINT2* inhibits HGF activator, which prevents the formation of active hepatocyte growth factor, has been taken as a putative tumor suppressor [32]. Previous studies mainly focus on the methylation of *SPINT2* in HCC instead of its expression [33, 34]. Nevertheless, we have found that the expression level of *SPINT2* was significantly suppressed in HCC expression profiles. The pattern was consistent with that in cell renal cell carcinoma [32], which might indicate its potential application as a novel HCC suppressor.

*ECM1* encodes a soluble protein that is involved in endochondral bone formation, angiogenesis, and tumor biology. It interacts with a variety of extracellular and structural proteins, contributing to the maintenance of skin integrity and homeostasis [35]. The expression of *ECM1* is reported to be significantly up-regulated in HCC patients [24], however, the current analyses of expression profiles showed that expression of *ECM1* was suppressed in HCC patients and were confirmed using RT-PCR. The discrepancy revealed the complicated functions of *ECM1* in the oncogenesis and development of HCC.

## Conclusions

In short, the current study gave an explicit elucidation of dysregulated genes in HCC by the integrated analysis of microarray datasets in GEO database, the biological function of these genes was significantly enriched in cell cycle. The results of RT-PCR and TCGA validation were consistent with that of integrated analysis, indicating the high credibility of this integrated analysis method. In addition, our study showed that some genes could be potentially valuable in the clinical diagnosis (such as *ASPM, NEK2* and *CCT3*) and anticancer therapy (such as *DCN, RND3*) for HCC. Our study improved the understanding of the transcriptome status of HCC, and might shed a light on the further investigation on the mechanisms of HCC.

## Additional file

Additional file 1:
**Table S1.** Detail information of primers. **Table S2.** Information of the most significantly up-regulated or down-regulated DEGs in HCC. **Table S3.** The expression values of 10 genes on all 5 HCC cases. (DOC 89 kb)

## Abbreviations

BioGRID: Biological General Repository for Interaction Datasets
DEG: Differential expressed genes
GEO: Gene Expression Omnibus
GO: Gene ontology
HCC: Hepatocellular carcinomas
KEGG: Kyoto Encyclopedia of Genes and Genomes
MATLAB: MATrixLABoratory
PPI: Protein-protein interaction
RT-PCR: Real time polymerase chain reaction
