# Supplementary material for: A transcriptome profile in hepatocellular carcinomas based on integrated analysis of microarray studies
Source: Diagn Pathol. 2017 Jan 13;12:4. doi: 10.1186/s13000-016-0596-x (PMC5237304; doi:10.1186/s13000-016-0596-x)
Supplement: Additional file 1: — Table S1. Detail information of primers. Table S2. Information of the most significantly up-regulated or down-regulated DEGs in HCC. Table S3. The expression values of 10 genes on all 5 HCC cases. (DOC 89 kb) [file 13000_2016_596_MOESM1_ESM.doc]

**Table S1 Detail information of primers**

| Gene ID |  | Sequence (3’-5’) | Production size (bp) |
| --- | --- | --- | --- |
| *CAP 2* | Forward | AGTCACACTCCAAGTCCCACATCTC | 71 |
|  | Backward | TCCAACTCCAACACTGGGGCA |  |
| *ASPM* | Forward | TTACCTGATCCACCATTACCATCCT | 99 |
|  | Backward | ACCACTGAACCAGTTTGCGTACATTC |  |
| *SNRPE* | Forward | TTACAAAATAGATCGCGGATTCAGG | 158 |
|  | Backward | ATCCGACCCAGTTGTTTTCTTGAC |  |
| *CCT3* | Forward | CCAGCACCATCCGTCTACTTACCT | 84 |
|  | Backward | CCGTCTCACCATTTACACCCCAG |  |
| *NEK2* | Forward | GGCTGCTTGCTGTATGAGTTATGTG | 271 |
|  | Backward | GCTCTCCTAATTGTCGCCCTCTT |  |
| *CLEC4M* | Forward | AACAGTGGAGAACCCAACAATAGCG | 102 |
|  | Backward | GCAGATCCAGTAATTGTCAACGTCA |  |
| *SPINT2* | Forward | TGGTACTTTGACGTGGAGAGGAACT | 85 |
|  | Backward | CCTCAGAGCGGTAGCTGTTCTTATT |  |
| *DCN* | Forward | GTAGTTGGATCAAGTGACTTCTGCCC | 81 |
|  | Backward | GTTGCTGAAAAGACTCACACCCGA |  |
| *ECM1* | Forward | TCGGGCTCCTTACCCCAACTAT | 113 |
|  | Backward | TTATGCTTGGTGAGAACTCTTTGGT |  |
| *RND3* | Forward | TTTACAGTCGGAAAATAGCGTCAGA | 110 |
|  | Backward | GCTTTGTGGCTCTCTGTGATTTGTT |  |
| *Actin* | Forward | ACTTAGTTGCGTTACACCCTT | 156 |
|  | Backward | GTCACCTTCACCGTTCCA |  |

**Table S2 Information of the most significantly DEGs in up-regulation or down-regulation**

|  | **Gene Symbol** | **Pubmed ID** | **Function** | **P-value** | **Effect Size** |
| --- | --- | --- | --- | --- | --- |
| Up-regulated | *NDC80* | 23474708 | Required for chromosome segregation and spindle checkpoint activity; required for kinetochore integrity and the organizationof stable microtubule binding sites in the outer plate of the kinetochore | 0 | -1.34 |
|  | *CAP2* | 17000669 | May have a regulatory bifunctional role | 0 | -1.60 |
|  | *ASPM* | 18676753 | Probable role in mitotic spindle regulation and coordination of mitotic processes; may have a preferential role in regulating neurogenesis | 0 | -1.46 |
|  | *CNIH4* | 17367606 | N/A | 0 | -1.91 |
|  | *SNRPE* | 21688285 | Core component of the spliceosomal U1, U2, U4 and U5 small nuclear ribonucleoproteins (snRNPs), the building blocks of the spliceosome; may indirectly play a role in hair development | 0 | -1.31 |
|  | *TBCE* | 18571892 | Tubulin-folding protein; involved in the second step of the tubulin folding pathway; involved in regulation of tubulin heterodimer dissociation | 0 | -1.73 |
|  | *CCT3* | 12079511 | Molecular chaperone; assists the folding of proteins upon ATP hydrolysis; may play a role in the assembly of BBSome, a complex involved in ciliogenesis regulating transports vesicles to the cilia; play a role, in vitro, in the folding of actin and tubulin | 0 | -2.19 |
|  | *SLC38A6* | 24752331 | Probable sodium-dependent amino acid/proton antiporter | 0 | -1.53 |
|  | *CDH13* | 12374686 | May thus contribute to the sorting of heterogeneous cell types. may act as a negative regulator of neural cell growth | 3.93E-14 | -1.63 |
|  | *NEK2* | 19136513 | Involved in the control of centrosome separation and bipolar spindle formation in mitotic cells and chromatin condensation in meiotic cells; regulates centrosome separation; involved in regulation of mitotic checkpoint protein complex; plays an active role in chromatin condensation during the first meiotic division | 0 | -1.81 |
| Down-regulated | *CLEC4M* | 16894195 | Probable pathogen-recognition receptor involved in peripheral immune surveillance in liver; may mediate the endocytosis of pathogens which are subsequently degraded in lysosomal compartments; probably recognizes in a calcium-dependent manner high mannose N-linked oligosaccharides | 0 | 2.71 |
|  | *SPINT2* | 19107935 | Inhibitor of HGF activator; also inhibits plasmin, plasma and tissue kallikrein, and factor XIa | 0 | 2.51 |
|  | *ADAMTS13* | 21876190 | Cleaves the vWF multimers in plasma into smaller forms | 0 | 1.57 |
|  | *DCN* | 23754492 24361483 | May affect the rate of fibrils formation | 0 | 2.00 |
|  | *ECM1* | 21128013 | Involved in endochondral bone formation as negative regulator of bone mineralization; stimulates the proliferation of endothelial cells and promotes angiogenesis; inhibits MMP9 proteolytic activity | 0 | 3.52 |
|  | *KAZN* | 16894195 | May be involved in the interplay between adherens junctions and desmosomes; the function in the nucleus is not known | 0 | 1.54 |
|  | *SH3YL1* | 19107935 | N/A | 0 | 1.79 |
|  | *RBMS3* | 21876190 | Binds poly(A) and poly(U) oligoribonucleotides | 0 | 2.41 |
|  | *RND3* | 23754492 24361483 | Binds GTP but lacks intrinsic GTPase activity and is resistant to Rho-specific GTPase-activating proteins | 0 | 2.06 |
|  | *CLEC4M* | 21128013 | Probable pathogen-recognition receptor involved in peripheral immune surveillance in liver; may mediate the endocytosis of pathogens; probably recognizes in a calcium-dependent manner high mannose N-linked oligosaccharides. | 0 | 2.05 |

**Table S3 The expression values of 10 genes on all 5 HCC cases**

| **Genes** | **2-△△ct** | | | | | **Mean ± SD** | **P value** |
| --- | --- | --- | --- | --- | --- | --- | --- |
|  | **No. 1** | **No. 2** | **No. 3** | **No. 4** | **No. 5** |  |  |
| CAP2 | 1.7278 | 4.6943 | 2.1945 | 1.5389 | 2.182 | 2.4675±1.2771 | ＜0.01 |
| ASPM | 3.4862 | 13.4255 | 3.5365 | 5.193 | 5.9216 | 6.3126±4.1138 | ＜0.01 |
| SNRPE | 1.572 | 2.2365 | 1.3748 | 3.2606 | 1.372 | 1.9632±0.8073 | ＜0.01 |
| CCT3 | 3.4485 | 1.5819 | 2.0152 | 2.5576 | 2.6944 | 2.4595±0.709 | ＜0.01 |
| NEK2 | 4.3911 | 14.6309 | 3.8032 | 2.0224 | 11.3744 | 7.2444±5.4514 | ＜0.01 |
| CLEC4M | 0.0187 | 0.0056 | 0.0296 | 0.4991 | 0.0015 | 0.1109±0.2173 | ＜0.01 |
| SPINT2 | 0.2964 | 0.0142 | 0.0685 | 0.1552 | 0.0248 | 0.1118±0.1172 | ＜0.01 |
| DCN | 0.4248 | 0.0189 | 0.1194 | 0.1281 | 0.0255 | 0.1433±0.1654 | ＜0.01 |
| ECMI | 0.3519 | 0.0452 | 0.5673 | 0.741 | 0.0329 | 0.3477±0.3137 | ＜0.01 |
| RND3 | 0.1426 | 0.0686 | 0.757 | 0.6867 | 0.0726 | 0.3455±0.3457 | ＜0.01 |
